# Supplementary material for: Causal Relationship between Aging and Anorexia Nervosa: A White-Matter-Microstructure-Mediated Mendelian Randomization Analysis
Source: Biomedicines. 2024 Aug 16;12(8):1874. doi: 10.3390/biomedicines12081874 (PMC11351342; doi:10.3390/biomedicines12081874)
Supplement: Supplementary file 1 [file biomedicines-12-01874-s001.zip › Supplementary Figure/Figure S4/Singe SNP plot of MR analysis for TL to ubm-b-1493.pdf]

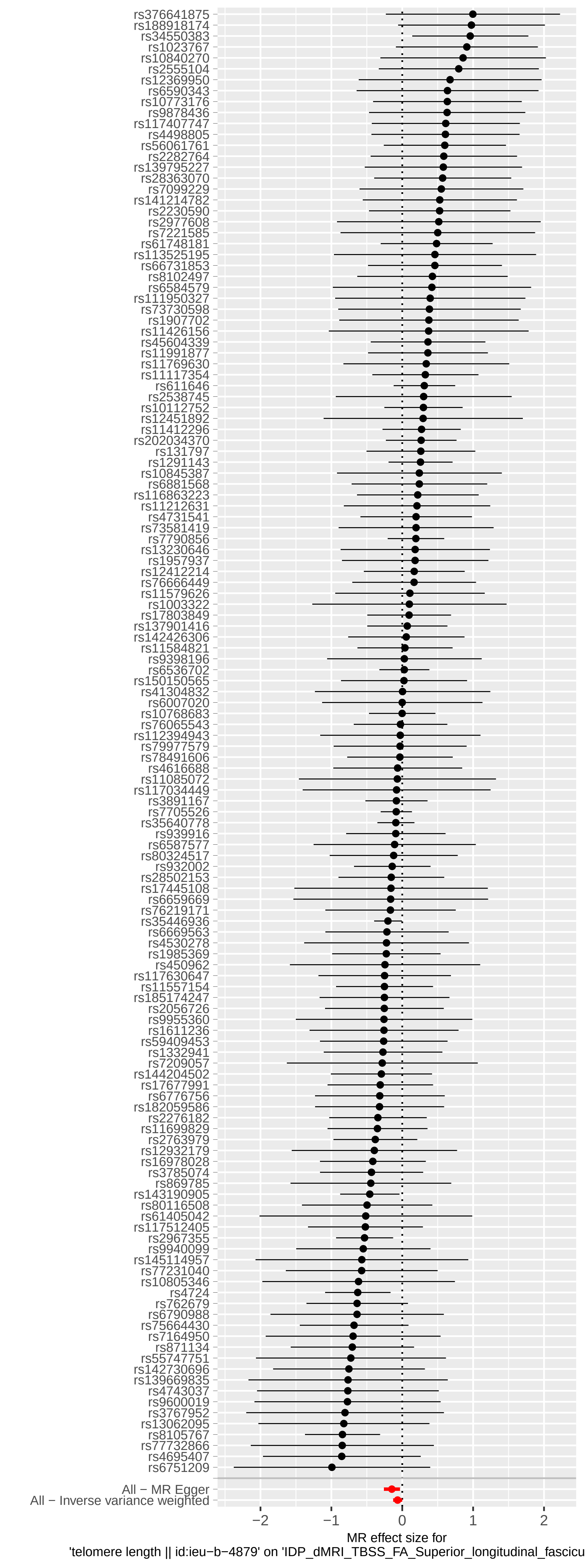
rs376641875
rs188918174
rs34550383
rs1023767
rs10840270
rs2555104
rs12369950
rs6590343
rs10773176
rs9878436
rs117407747
rs4498805
rs56061761
rs2282764
rs139795227
rs28363070
rs7099229
rs141214782
rs2230590
rs2977608
rs7221585
rs61748181
rs113525195
rs66731853
rs8102497
rs6584579
rs111950327
rs73730598
rs1907702
rs11426156
rs45604339
rs11991877
rs11769630
rs11117354
rs611646
rs2538745
rs10112752
rs12451892
rs11412296
rs202034370
rs131797
rs1291143
rs10845387
rs6881568
rs116863223
rs11212631
rs4731541
rs73581419
rs7790856
rs13230646
rs1957937
rs12412214
rs76666449
rs11579626
rs1003322
rs17803849
rs137901416
rs142426306
rs11584821
rs9398196
rs6536702
rs150150565
rs41304832
rs6007020
rs10768683
rs76065543
rs112394943
rs79977579
rs78491606
rs4616688
rs11085072
rs117034449
rs3891167
rs7705526
rs35640778
rs939916
rs6587577
rs80324517
rs932002
rs28502153
rs17445108
rs6659669
rs76219171
rs35446936
rs6669563
rs4530278
rs1985369
rs450962
rs117630647
rs11557154
rs185174247
rs2056726
rs9955360
rs1611236
rs59409453
rs1332941
rs7209057
rs144204502
rs17677991
rs6776756
rs182059586
rs2276182
rs11699829
rs2763979
rs12932179
rs16978028
rs3785074
rs869785
rs143190905
rs80116508
rs61405042
rs117512405
rs2967355
rs9940099
rs145114957
rs77231040
rs10805346
rs4724
rs762679
rs6790988
rs75664430
rs7164950
rs871134
rs55747751
rs142730696
rs139669835
rs4743037
rs9600019
rs3767952
rs13062095
rs8105767
rs77732866
rs4695407
rs6751209
All − MR Egger
All − Inverse variance weighted
−2
−1
0
1
2
MR effect size for
'telomere length || id:ieu−b−4879' on 'IDP_dMRI_TBSS_FA_Superior_longitudinal_fascicu
